# IGF2BP3 Modulates mRNA Splicing and Stability to Promote Trophoblast Progression via Interaction with PDE3A and Suppression by miR-196a-5p in Preeclampsia

**DOI:** 10.3390/biomedicines13061268

**Published:** 2025-05-22

**Authors:** Chunyan Li, Pingpo Ming, Cuifang Fan, Jiao Chen, Jing Yang

**Affiliations:** 1 Department of Obstetrics, Renmin Hospital of Wuhan University, Wuhan 430060, China; chunyanlidoc@gmail.com (C.L.); fancuifang@whu.edu.cncom (C.F.); 2 Department of Oncology, Renmin Hospital of Wuhan University, Wuhan 430060, China; pingpomingdoc@gmail.com; 3 Reproductive Medicine Center, Renmin Hospital of Wuhan University, Wuhan 430060, China; drchenjiao@whu.edu.cn

**Keywords:** preeclampsia, alternative splicing, IGF2BP3, PDE3A, miR-196a-5p

## Abstract

**Background:** Preeclampsia (PE) is a pregnancy-specific disorder and a leading cause of maternal and fetal morbidity and mortality. Impaired trophoblast invasion is a hallmark of PE, and alternative splicing (AS) is crucial for trophoblast differentiation and placental development. However, the exact mechanisms of AS in PE remain poorly understood. **Methods:** To elucidate AS-mediated regulatory pathways in PE, a total of 38 fresh-frozen placental samples, including 13 pre-eclampsia samples and 25 normal control samples, were collected from Renmin Hospital of Wuhan University between 1 February and 30 July 2022. We performed transcriptome sequencing of seven PE and seven normal placentas to identify differentially spliced events. After quality control and adapter trimming, raw sequencing reads were aligned to the human reference genome using STAR. Differential exon usage was analyzed using DEXSeq (version 1.36.0), and exons with an adjusted *p*-value < 0.05 and a fold change greater than 2 or less than 0.5 were considered significantly differentially spliced. Functional assays, including CCK8, colony formation, and cell cycle analyses, were conducted to assess trophoblast proliferation, whereas wound healing and Transwell assays were used to evaluate trophoblast migration and invasion using the HTR-8/SVneo cell line. RNA immunoprecipitation sequencing (RIP-seq) and RNA stability assays were employed to investigate mRNA interactions and stability. **Results:** Insulin-like growth factor 2 mRNA-binding protein 3 (IGF2BP3) emerged as a key RNA-binding protein associated with alternative splicing regulation, intersecting both AS-related candidate genes and known splicing factors, although it is not a classical splicing factor itself. IGF2BP3 overexpression markedly enhanced HTR-8/SVneo trophoblast proliferation, migration, and invasion while suppressing ROS activation. RNA-seq, RIP-seq, and RNA stability assays revealed that IGF2BP3 directly interacts with and enhances the stability of PDE3A mRNA. Functional rescue experiments confirmed that PDE3A knockdown partially abrogated IGF2BP3-mediated trophoblast progression. Furthermore, miR-196a-5p was identified as a negative regulator of IGF2BP3 via miRNA inhibitor/mimic transfection, qRT-PCR, and functional assays, confirming that miR-196a-5p overexpression downregulates IGF2BP3, thereby impairing trophoblast migration and proliferation. Notably, restoring IGF2BP3 expression reversed these inhibitory effects. **Conclusions:** Our findings reveal a previously unrecognized regulatory axis in PE in which miR-196a-5p suppresses IGF2BP3 expression, leading to PDE3A mRNA destabilization and impaired trophoblast function. This study offers mechanistic insights into PE pathogenesis and identifies IGF2BP3 as a potential therapeutic target.

## 1. Introduction

Preeclampsia (PE) is a hypertensive disorder unique to pregnancy and is characterized by new-onset hypertension (≥140/90 mmHg) and proteinuria (≥300 mg/24 h) or other organ dysfunction after 20 weeks of gestation [1]. It remains one of the three causes of maternal and fetal morbidity and mortality worldwide. In addition to hypertension and renal dysfunction, PE can result in severe complications, including endothelial dysfunction, cardiovascular abnormalities, and multiorgan failure, posing significant risks to both mothers and fetuses [2]. The pathogenesis of PE is multifactorial, with placental insufficiency, hypoxia, excessive oxidative stress, and trophoblast dysfunction playing central roles. Aberrant trophoblast invasion and impaired migration contribute to defective placentation, ultimately leading to the clinical manifestations of PE [3]. However, the molecular mechanisms governing trophoblast dysfunction remain incompletely understood.

RNA-binding proteins (RBPs) are key regulators of posttranscriptional gene expression and influence mRNA splicing, stability, transport, and translation [4]. Dysregulated RBP activity is implicated in various pathological conditions, including cancer and cardiovascular diseases [5]. Alternative splicing (AS) is a post-transcriptional mechanism by which a single gene can generate multiple mRNA isoforms through the selective inclusion or exclusion of specific exons or introns. This process significantly expands the proteomic diversity without increasing the number of genes, allowing cells to fine-tune gene expression in response to developmental cues and environmental stimuli [6]. Increasing evidence suggests that alternative splicing (AS) plays a critical role in trophoblast differentiation and placental development [7]. Aberrant RNA processing, including altered AS, has been associated with PE, but the underlying regulatory mechanisms remain elusive. Identifying key RBPs involved in PE-related AS events may provide novel insights into disease pathogenesis and therapeutic targets.

Insulin-like growth factor 2 mRNA-binding protein 3 (IGF2BP3), an RBP known for its role in RNA metabolism, which was first demonstrated in neuronal development of mouse embryos [8], has been extensively studied in cancer, where it promotes proliferation, adhesion, and invasion [9]. IGF2BP3 functions as an m6A reader, stabilizing oncogenic transcripts such as CCND1 and VEGF to increase tumor progression [10]. Additionally, IGF2BP3 modulates AS events, including those of PKM, contributing to metabolic reprogramming in cancer [11,12]. While its role in malignancies is well established, the function of IGF2BP3 in trophoblast biology and PE remains poorly defined. Notably, IGF2BP3 expression is significantly downregulated in PE placentas but is elevated in early pregnancy trophoblasts, suggesting a potential role in placental development [13]. Knockdown of IGF2BP3 in trophoblast cells impairs migration and invasion; however, its involvement in AS regulation in PE has not been fully explored.

In this study, we investigated the regulatory role of IGF2BP3 in PE, focusing on its function in AS and mRNA stability. Using RNA sequencing (RNA-seq) and RNA immunoprecipitation sequencing (RIP-seq), we identified PDE3A as a key IGF2BP3 target. IGF2BP3 directly stabilizes PDE3A mRNA, modulating its splicing patterns and expression in trophoblast cells. Functional assays demonstrated that IGF2BP3 overexpression promoted trophoblast proliferation, migration, and invasion, whereas PDE3A knockdown partially reversed these effects. Moreover, we identified miR-196a-5p as a negative regulator of IGF2BP3, further modulating the IGF2BP3-PDE3A axis. Collectively, our findings reveal a novel miR-196a-5p/IGF2BP3/PDE3A regulatory pathway involved in trophoblast function, providing mechanistic insights into PE pathogenesis and identifying potential therapeutic targets.

## 2. Materials and Methods

### 2.1. Tissue Collection

A total of 38 fresh-frozen placental samples, including 13 from patients with preeclampsia (PE) and 25 from normotensive controls, were collected at Renmin Hospital of Wuhan University between 1 February and 30 July 2022. The gestational age at delivery ranged from 29^+3^ to 37^+1^ weeks in the PE group and from 37 to 39^+2^ weeks in the control group. Among the 13 PE cases, 6 were just diagnosed with preeclampsia and 7 with severe preeclampsia (sPE). All the tissue samples were immediately snap-frozen and stored at −80 °C until further analysis. This study was approved by the Ethics Committee of Renmin Hospital of Wuhan University (Approval No. WDRY2022-K103), and written informed consent was obtained from all participants.

### 2.2. RNA Extraction, Sequencing, and Data Analysis

To comprehensively characterize AS events in preeclampsia and their underlying regulation, we performed transcriptomic profiling on two sample types. Placental tissues from 7 sPE patients and 7 normotensive controls were used to identify clinically relevant AS events. In parallel, the HTR-8/SVneo trophoblast cell line was used to conduct RNA-seq and functional experiments under defined experimental conditions, enabling mechanistic validation of candidate regulators and splicing events.

Total RNA was extracted from 14 fresh-frozen placental tissues, including 7 from sPE patients and 7 from normotensive controls, using TRIzol Reagent (Invitrogen, Carlsbad, CA, USA). RNA quality and concentration were evaluated using NanoDrop™ One C and Qubit™ 3.0 fluorometer (Thermo Fisher Scientific, Waltham, MA, USA), and integrity was assessed via 1.5% agarose gel electrophoresis.

Total RNA was extracted from HTR-8/SVneo cells via TRIzol Reagent (Invitrogen, Cat. No. 15596026) and subsequently treated with DNase I to remove genomic DNA contamination. RNA purity was confirmed by measuring the A260/A280 ratio (NanoDrop™ One C, Thermo Fisher Scientific), and RNA integrity was verified via 1.5% agarose gel electrophoresis. The RNA concentrations were quantified via a Qubit™ RNA Broad Range Assay Kit (Life Technologies, Carlsbad, CA, USA, Q10210) on a Qubit™ 3.0 fluorometer. The quality and integrity of total RNA were assessed using an Agilent 2100 Bioanalyzer (Agilent Technologies, Santa Clara, CA, USA). Samples with RIN values greater than 7.0 were selected for RNA-seq library construction and downstream analyses.

For library preparation, 2 μg of total RNA was used to construct stranded mRNA libraries with the KCTM Stranded mRNA Library Prep Kit for Illumina (Cat. No. DR08402, Wuhan Seqhealth Co., Ltd., Wuhan, China) following the manufacturer’s protocol. PCR products in the range of 200–500 bp were enriched and quantified before sequencing on an Illumina NovaSeq 6000 platform (PE150) (San Diego, CA, USA).

Raw sequencing reads were initially filtered by removing those containing >2 ambiguous bases (N) and trimming adaptor sequences and low-quality bases via the FASTX-Toolkit (version 0.0.13). Reads shorter than 16 nt were discarded [14]. Clean reads were aligned to the GRCh38 reference genome via HISAT2, allowing up to four mismatches [15]. Only uniquely mapped reads were retained for subsequent analyses.

Gene expression levels were quantified as fragments per kilobase of transcript per million mapped reads (FPKM) [16]. For differentially expressed gene analysis, the R Bioconductor package DESeq214 was used to screen out the DEGs. Adjusted *p* value < 0.05 (Benjamini–Hochberg) and |log2 fold change| > 1 were used as cutoffs [17].

The alternative splicing events (ASEs) and regulated alternative splicing events (RASEs) between the samples were defined and quantified via the ABL as pipeline. In brief, ABL detection of ten types of ASEs was based on splice junction reads, including exon skipping (ES), alternative 5′ splice site (A5SS), alternative 3’s splice site (A3SS), mutually exclusive exons (MXE), mutually exclusive 5′UTRs (5pMXE), mutually exclusive 3′UTRs (3pMXE), cassette exons, A3SS&ES and A5SS&ES. To assess RBP -regulated ASE, Student’s *t* test was performed to evaluate the significance of the ratio alteration of AS events. Those events that were significant at the *p* value cutoff corresponding to a false discovery rate cutoff of 5% were considered RBP-regulated ASEs.

Functional enrichment analysis of DEGs was performed via the KOBAS 2.0 server [18]. Gene Ontology (GO) terms and KEGG pathway enrichment were determined via a hypergeometric test with Benjamini-Hochberg false discovery rate (FDR) correction. Data visualization and statistical analyses were carried out via tools as below. Differential alternative splicing events were analyzed using rMATS (v4.1.0), while differential gene expression analysis was performed using DESeq2 (v1.36.0). GO and KEGG enrichment analyses were conducted with clusterProfiler (v4.2.2). Sashimi plots were visualized using IGV (v2.12.3). PCA and volcano plots were generated in R using ggplot2 and EnhancedVolcano packages, respectively. Motif analysis was carried out with the HOMER tool (v4.11). Heatmaps were plotted using ComplexHeatmap (v2.12.0), and Venn diagrams were created with the VennDiagram R package.

### 2.3. Coimmunoprecipitation and Library Preparation

HTR-8/SVneo cells were exposed once to ultraviolet C (UVC) radiation at 254 nm with a total energy dose of 400 mJ/cm^2^. After irradiation, cells were immediately lysed on ice using a chilled buffer composed of 1 × PBS, 0.1% SDS, 0.5% NP-40, and 0.5% sodium deoxycholate, supplemented with RNase inhibitor (200 U/mL, Takara) and a protease inhibitor cocktail (Roche, Basel, Switzerland). The lysates were kept on ice for 30 min. Cellular debris was cleared by centrifugation at 10,000 rpm for 10 min at 4 °C.

To degrade contaminating DNA, RQ I DNase (Promega, Madison, WI, USA, 1 U/μL) was added to the supernatant to reach a final concentration of 0.05 U/μL, followed by incubation in a 37 °C water bath for 30 min. The enzymatic reaction was then halted by the addition of a stop solution. Samples were vortexed thoroughly and centrifuged at 13,000× *g* for 20 min at 4 °C to remove any residual debris.

Subsequently, RNA was fragmented using micrococcal nuclease (MNase, Thermo Scientific, Waltham, MA, USA). For the immunoprecipitation step, the resulting supernatant was incubated overnight at 4 °C with 10 μg of anti-Flag antibody (Proteintech, Rosemont, IL, USA, FNab03155) and a control IgG (CST, Danvers, MA, USA, 5873S). Antibody–protein complexes were then captured by incubating with protein A/G-conjugated Dynabeads (Thermo Scientific) for 2 h at 4 °C.

After bead capture, magnetic separation was performed, and the beads were washed sequentially using three buffers: the original lysis buffer, a high-salt wash solution (250 mM Tris-HCl pH 7.4, 750 mM NaCl, 10 mM EDTA, 0.1% SDS, 0.5% NP-40, 0.5% sodium deoxycholate), and PNK buffer (50 mM Tris, 20 mM EGTA, 0.5% NP-40), each applied twice.

The beads were then resuspended in elution buffer (50 mM Tris-HCl pH 8.0, 10 mM EDTA, 1% SDS) and incubated at 70 °C for 20 min to release the RNA–protein complexes. After vortexing, the magnetic beads were removed and the supernatant transferred to a clean microcentrifuge tube. Both input samples (non-immunoprecipitated) and the immunoprecipitated complexes were treated with Proteinase K (Roche) at a final concentration of 1.2 mg/mL and incubated at 55 °C for 2 h.

RNA was then extracted using Trizol reagent (Life Technologies). Subsequent library preparation was carried out using the KAPA RNA Hyper Prep Kit (KAPA, Nairobi, Kenya, KK8541) in accordance with the manufacturer’s guidelines. Finally, libraries were sequenced on the Illumina NovaSeq platform using a 150 bp paired-end read configuration

### 2.4. Data Analysis

After reads were aligned onto the genome with HISAT2, the unique comparison on the genome was finally obtained, and the comparison result of PCR duplicate was removed. And then two software programs, Piranha(version 1.2.1) and ABLIRC(version 0.2),were used to perform peak calling. Piranha has been described elsewhere [19]. “ABLIRC” strategy was used to identify the binding regions of GRCh38 on genome. The peak calling procedure was carried out as follows: the entire genome was scanned using a sliding window approach, with both window size and step length set to 5 bp, starting from the beginning of each chromosome. A region was considered a candidate peak if the read depth in the initial window exceeded 2.5 times the baseline for eight consecutive windows, or if the median depth surpassed 50. The termination of a peak was defined when the depth across the eight-window segment dropped below 4% of the peak’s maximum read depth. To assess statistical significance, a randomization process was performed where reads were shuffled and reassigned to genes 500 times. The frequency distribution of peak depths from these simulations was used to calculate significance, retaining peaks with either a *p*-value less than 0.05 or a maximum depth ≥10.

Next, differential enrichment analysis was conducted using input samples as controls. Peaks with immunoprecipitated (IP) read counts at least fourfold higher than those in the input (this threshold was adjustable) were defined as enriched binding sites. These final peaks were used to identify target genes of the IP protein. Furthermore, the corresponding binding motifs were identified using the HOMER (version 4.11.1) software suite. The target genes of IP were finally determined by the peaks and the binding motifs of IP protein were called by HOMER software [20].

### 2.5. Immunohistochemistry

Placental samples obtained from patients with preeclampsia were promptly fixed in paraformaldehyde and embedded in paraffin. The embedded tissues were then subjected to dehydration through a graded ethanol series and subsequently cleared using xylene. After rehydration, sections were rinsed three times with Tris-buffered saline (TBS) and antigen retrieval was performed using sodium citrate buffer.

To block non-specific binding, sections were incubated with 5% bovine serum albumin (BSA) for 30 min. This was followed by overnight incubation at 4 °C with a biotin-labeled primary antibody specific to IGF2BP3. Afterward, a streptavidin-conjugated secondary antibody was applied for 30 min at ambient temperature. Immunoreactivity was visualized using 3,3′-diaminobenzidine (DAB; ZLI-9019, Beijing, China), and nuclei were counterstained with hematoxylin. Images were captured using an Olympus DP72 light microscope.

### 2.6. Cell Culture and Transfections

HTR-8/SVneo cells (Cell Research Science & Technology Co., Ltd., Shanghai, China) were maintained in RPMI 1640 medium supplemented with 10% fetal bovine serum (FBS), 100 U/mL penicillin, and 100 μg/mL streptomycin. Cells were incubated at 37 °C in a humidified environment with 5% CO_2_. For overexpression experiments, cells were transfected with the pcDNA3.1-3×Flag-IGF2BP3 plasmid (Youbio Biotech, Changsha, China) using Lipofectamine 2000 (Invitrogen) following the manufacturer’s guidelines. After 48 h of transfection, cells were collected for RT-qPCR and Western blot analyses.

### 2.7. Cell Proliferation and Colony Formation Assays

For proliferation assays, 5 × 10^3^ HTR-8/SVneo cells were plated into 96-well plates. Cell viability was evaluated every 24 h using a CCK8 assay, with 10 μL reagent added per well and incubated at 37 °C for 1 h. Absorbance at 450 nm was measured using a microplate reader. For colony formation analysis, 1 × 10^3^ cells were seeded in 6-well plates and cultured for 15 days. Colonies were fixed with 4% paraformaldehyde, stained with hematoxylin, and examined using a DP72 microscope (Olympus, Tokyo, Japan).

### 2.8. Cell Cycle Analysis

Cells were harvested, washed with PBS, and fixed in cold 70% ethanol for 30 min at 4 °C. Following fixation, cells were washed, resuspended in RNase A solution, and incubated at 37 °C for 30 min. Propidium iodide (PI) staining was performed, and cell cycle distribution was analyzed using a BD FACSCalibur Flow Cytometer (Becton Dickinson Biosciences, San Jose, CA, USA). Data were processed with ModFit LT 5.0 software.

### 2.9. Scratch Wound and Transwell Migration Assays

For the scratch wound assay, HTR-8/SVneo cells were plated in 6-well dishes and cultured overnight. A straight scratch was generated using a 100 μL pipette tip, and the cells were subsequently incubated for 24 h. After PBS washing, the wound area was stained with crystal violet and imaged. The extent of wound closure was analyzed using ImageJ software (version 1.53k) (NIH).

For the Transwell migration assay, cells were placed in the upper compartment of a Matrigel-coated Transwell insert filled with serum-free RPMI 1640 medium. The lower compartment contained RPMI 1640 medium supplemented with 10% FBS to act as a chemoattractant. After 24 h, non-migratory cells on the upper surface were gently removed with a cotton swab. The migrated cells on the lower surface were then fixed with 4% paraformaldehyde, stained with crystal violet, and visualized under an inverted microscope.

### 2.10. Reactive Oxygen Species (ROS) Activity

To assess ROS production, HTR-8/SVneo cells were incubated with 5 μM C11-BODIPY 581/591 in 5% FBS-PBS at 37 °C for 1 h. After washing with PBS, fluorescence was measured using a BD FACSCalibur Flow Cytometer (BD Biosicences, Franklin Lakes, NJ, USA) equipped with a 561 nm laser and a 581/591 nm filter.

### 2.11. RNA Stability Assay

HTR-8/SVneo cells were treated with 5 μg/mL actinomycin D to inhibit transcription. Total RNA was extracted at 0, 20, 40, and 60 min after the addition of actinomycin D using TRIzol reagent (Invitrogen, Carlsbad, CA, USA). The mRNA levels of IGF2BP3 were quantified by RT-qPCR, and mRNA half-lives were calculated based on the decay curves using GraphPad Prism software (v9.0).

### 2.12. Quantitative Real-Time PCR (qRT-PCR)

Total RNA was isolated using TRIzol reagent (Invitrogen, Carlsbad, CA, USA) following the manufacturer’s protocol, including phenol-chloroform extraction and isopropanol precipitation steps. Complementary DNA (cDNA) was synthesized from 1 μg of RNA using the PrimeScript RT reagent Kit (Takara, Shiga, Japan) in accordance with the supplier’s instructions. Quantitative PCR was conducted on a Bio-Rad S1000 thermal cycler with Bestar SYBR Green RT-PCR Master Mix (Vazyme, Nanjing, China). Primer sequences are listed in Appendix A, Table A1. GAPDH served as an internal reference gene, and relative expression levels were determined by the 2^−ΔΔCT^ method. Statistical analysis was performed using paired Student’s *t*-test in GraphPad Prism software (San Diego, CA, USA).

### 2.13. Western Blot Analysis

Cells were lysed in ice-cold lysis buffer containing 1× PBS, 0.1% SDS, 0.5% NP-40, 0.5% sodium deoxycholate, and a protease inhibitor cocktail (Roche, Basel, Switzerland). The lysis was performed on ice for 30 min. Lysates were then mixed with SDS sample buffer and boiled at 95 °C for 10 min to denature proteins. Equal amounts of protein samples were separated by 10% SDS-PAGE and transferred onto polyvinylidene difluoride (PVDF) membranes. Membranes were blocked with 5% non-fat dry milk in TBST buffer (20 mM Tris-HCl, 150 mM NaCl, 0.1% Tween-20, pH 7.6) for 1 h at room temperature. Subsequently, membranes were incubated overnight at 4 °C with primary antibodies against FLAG (1:2000, Proteintech, 80010-1-RR) and GAPDH (1:1000, Goodhere, AB-P-R001). After washing, membranes were incubated with horseradish peroxidase (HRP)-conjugated secondary antibodies (1:10,000, Boster, BA1054, Schwanewede, Germany) for 1 h at room temperature. Protein bands were visualized using an enhanced chemiluminescence (ECL) detection kit (Bio-Rad, Hercules, CA, USA, 170506).

### 2.14. Dual-Luciferase Reporter Assay

The interaction between IGF2BP3 and miR-196a-5p was evaluated using a dual-luciferase reporter assay. The pmirGLO vector (Qingke Bio, Jinghai, China) was utilized to subclone the IGF2BP3 3′-UTR containing either the wild-type (WT) or mutant (Mut) miR-196a-5p binding sequence. HTR-8/SVneo cells were transfected with these plasmids along with miR-196a-5p mimic using Lipofectamine 2000. After a 48 h incubation, luciferase activity was quantified with the Dual-Luciferase Reporter Assay System (Promega, Madison, WI, USA) following the manufacturer’s instructions. All experiments were independently repeated in triplicate.

### 2.15. Statistical Analysis

Experimental results are expressed as mean ± standard deviation (SD) based on a minimum of three independent replicates. Statistical analyses were conducted using GraphPad Prism version 8.0 (GraphPad Software, San Diego, CA, USA). For comparisons, unpaired Student’s t-tests and one-way analysis of variance (ANOVA, San Francisco, CA, USA) were employed, along with suitable post hoc tests when applicable. A *p*-value less than 0.05 was considered indicative of statistical significance. Detailed descriptions of statistical methods and corresponding tests are provided in the respective figure legends.

## 3. Results

### 3.1. Differential Alternative Splicing of IGF2BP3 Occurs in Preeclampsia

**Figure 1:**
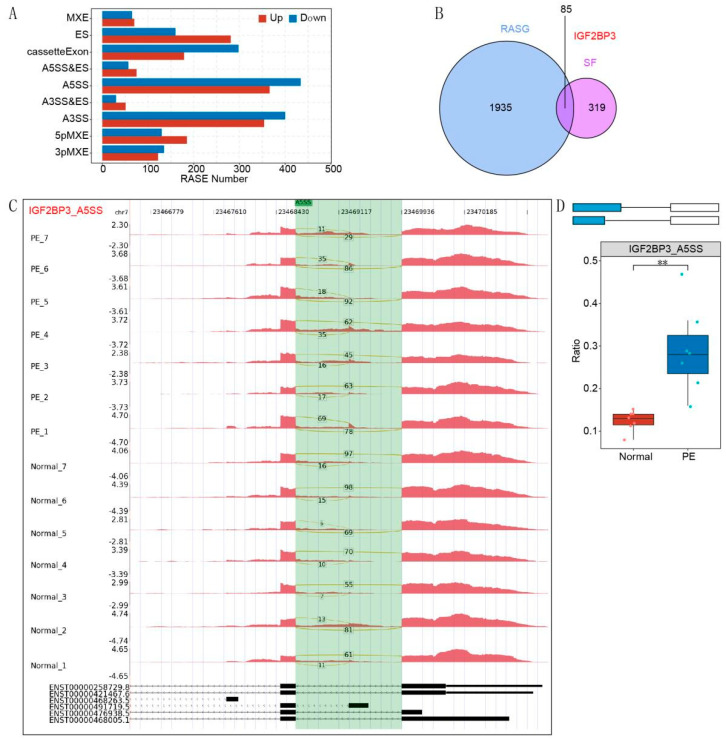
Differential alternative splicing of IGF2BP3 occurs in preeclampsia: (**A**) Bar graph illutrating the total number of significantly regulated alternative splicing events (RASEs). The X-axis represents RASE counts, while the Y-axis indicates different AS event types. (**B**) Venn diagram d-picting the number of regulated alternative splicing genes (RASGs) and associated splice factors (SFs). (**C**) Distribution of reads for the IGF2BP3 A5SS event, with the upper panel displaying read coverage and corresponding transcript structures shown below. (**D**) Box plot presenting the ratio of IGF2BP3 A5SS events. ** *p* < 0.01.

To investigate alternative splicing (AS) alterations associated with preeclampsia (PE), we performed transcriptome sequencing on placental samples from PE patients and normal controls. A comprehensive analysis revealed a total of significantly altered AS events (RASEs), as illustrated in Figure 1A. Subsequent candidate prioritization, based on an overlap between regulated alternative splicing genes (RASGs) and splice factors (SFs), identified IGF2BP3 as a gene of interest (Figure 1B). Notably, the distribution of reads for the alternative 5’ splice site (A5SS) event in IGF2BP3—one of the most frequently observed events in PE—differed markedly between PE and control samples (Figure 1C). Furthermore, quantitative analysis using a box plot confirmed that the ratio of the IGF2BP3 A5SS event was significantly elevated in PE placentas relative to normal controls (Figure 1D). Collectively, these results implicate aberrant IGF2BP3 splicing in the pathogenesis of PE and suggest its potential utility as a prognostic marker.

### 3.2. IGF2BP3 Promotes Proliferation and Migration of HTR-8/SVneo Trophoblast Cells

**Figure 2:**
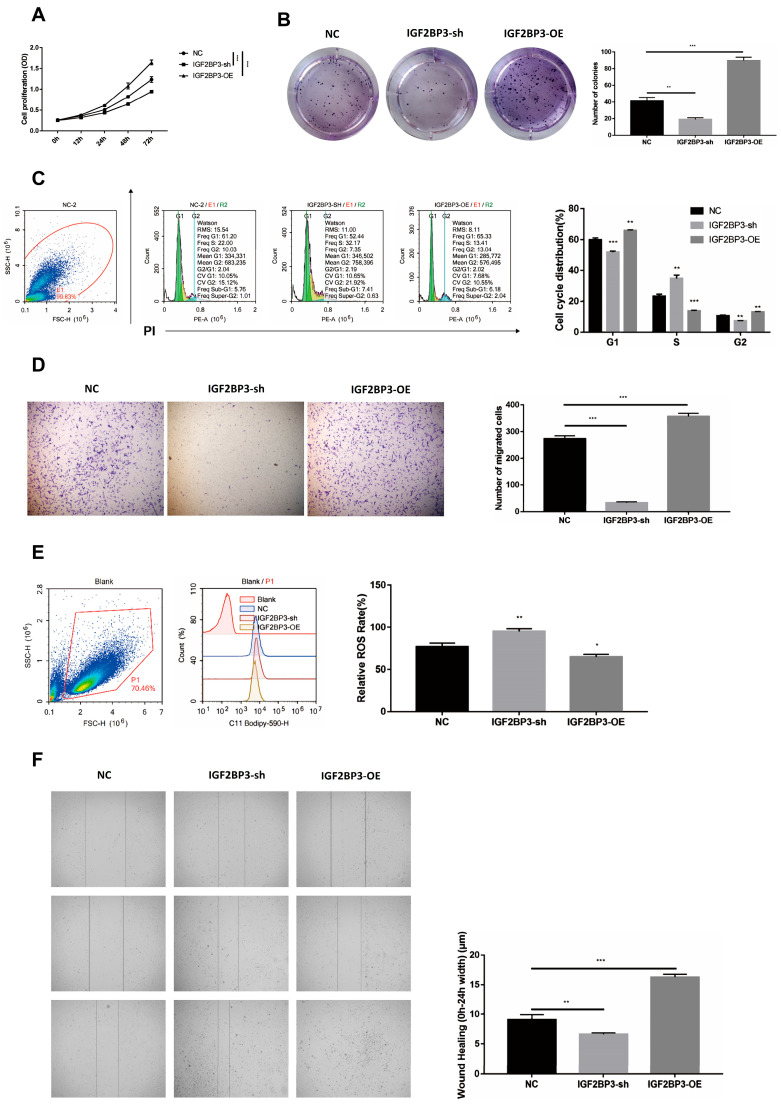
IGF2BP3 promotes proliferation and migration of HTR-8/SVneo trophoblast cells: (**A**) Cell proliferation was determined for HTR-8/SVneo trophoblast cells infected with overexpressing IGF2BP3, silencing IGF2BP3 or negative control by CCK8 assay. (**B**) Colony formation assays were performed with HTR-8/SVneo trophoblast cells infected with overexpressing IGF2BP3, silencing IGF2BP3 or negative control. (**C**) Cell cycle analysis was performed with HTR-8/SVneo trophoblast cells infected with overexpressing IGF2BP3, silencing IGF2BP3 or negative control. (**D**) Cell invasion measured by Transwell assay in HTR-8/SVneo trophoblast cells infected with overexpressing IGF2BP3, silencing IGF2BP3 or negative control. (**E**) ROS activity was measured by C11-BODIPY 581/591 intensity in HTR-8/SVneo trophoblast cells infected with overexpressing IGF2BP3, silencing IGF2BP3 or negative control. (**F**) Cell migration measured by wound healing assay in HTR-8/SVneo trophoblast cells infected with overexpressing IGF2BP3, silencing IGF2BP3 or negative control. Data at the right are presented as mean ± SEM. * *p* < 0.05, ** *p* < 0.01, *** *p* < 0.001.

Following the elucidation of IGF2BP3′s involvement in preeclampsia progression, its impact on HTR-8/SVneo trophoblast cell function was assessed by modulating its expression levels. Overexpression of IGF2BP3 significantly enhanced both proliferation and colony formation capabilities of these cells, as quantitatively demonstrated in Figure 2A,B. Cell cycle analyses further supported these findings, showing a marked decrease in the G1 phase duration in cells overexpressing IGF2BP3 compared to the control group, suggesting enhanced cell cycle progression (Figure 2C).

Furthermore, IGF2BP3 overexpression was found to suppress reactive oxygen species (ROS) activity, potentially mitigating oxidative stress within the trophoblast cells. Enhanced cellular motility was evident from wound healing and Transwell assays, indicating a significant increase in the migratory capacity of cells overexpressing IGF2BP3. Together, these results robustly demonstrate that IGF2BP3 is pivotal not only for enhancing trophoblast cell proliferation and reducing cell cycle duration but also for promoting cellular migration and reducing oxidative stress. This multifaceted role highlights IGF2BP3 as a critical molecular mediator in trophoblast biology and underscores its therapeutic potential in managing preeclampsia.

### 3.3. The IGF2BP3 Regulates Transcriptome in HTR-8/SVneo Trophoblast Cells

**Figure 3:**
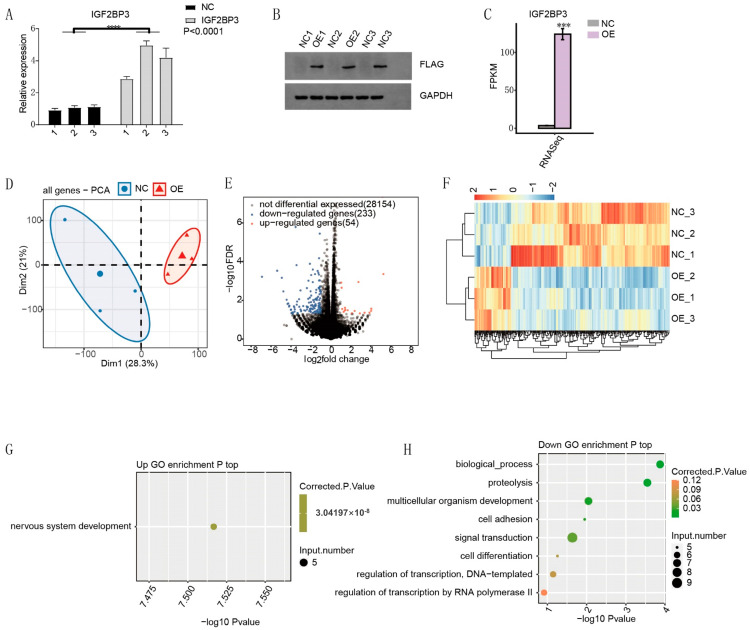
The IGF2BP3 regulates transcriptome in HTR8/SVneo trophoblast cells: (**A**) RT-qPCR analysis comparing control and IGF2BP3-overexpressing samples. (**B**) Western blot validation of IGF2BP3 overexpression. (**C**) IGF2BP3 mRNA expression levels in control and overexpression groups from RNA-seq data. (**D**) Principal component analysis (PCA) based on FPKM values of all identified genes. (**E**) Volcano plot displaying differential gene expression between IGF2BP3-overexpressing and control samples. (**F**) Heatmap showing hierarchical clustering of DEGs. (**G**) Bubble plot visualizing the top enriched GO terms among upregulated DEGs. (**H**) Bubble plot illustrating enriched GO terms for downregulated DEGs. All quantitative data are shown as mean ± SEM. Significance is denoted as *** *p* < 0.001, **** *p* < 0.0001.

To explore the transcriptional impact of IGF2BP3 overexpression in HTR-8/SVneo trophoblast cells, comprehensive RNA-seq analysis was conducted. Quantitative PCR and Western blot analyses confirmed a significant increase in IGF2BP3 expression, with RNA-seq further quantifying these changes (Figure 3A–C). Principal component analysis (PCA) based on FPKM values revealed distinct transcriptional profiles between the overexpressed and control groups (Figure 3D). Differential gene expression analysis, performed using edgeR, identified 54 upregulated and 233 downregulated genes, demonstrating extensive transcriptional regulation by IGF2BP3 (Figure 3E). Consistency across replicates was evident from the heatmap of IGF2BP3-regulated transcription (Figure 3F). Gene Ontology (GO) enrichment analysis of these differentially expressed genes (DEGs) revealed associations with nervous system development among upregulated genes and with biological processes and proteolysis among downregulated genes (Figure 3G,H).

### 3.4. IGF2BP3 Regulates Gene Alternative Splicing in HTR-8/SVneo Trophoblast Cells

**Figure 4:**
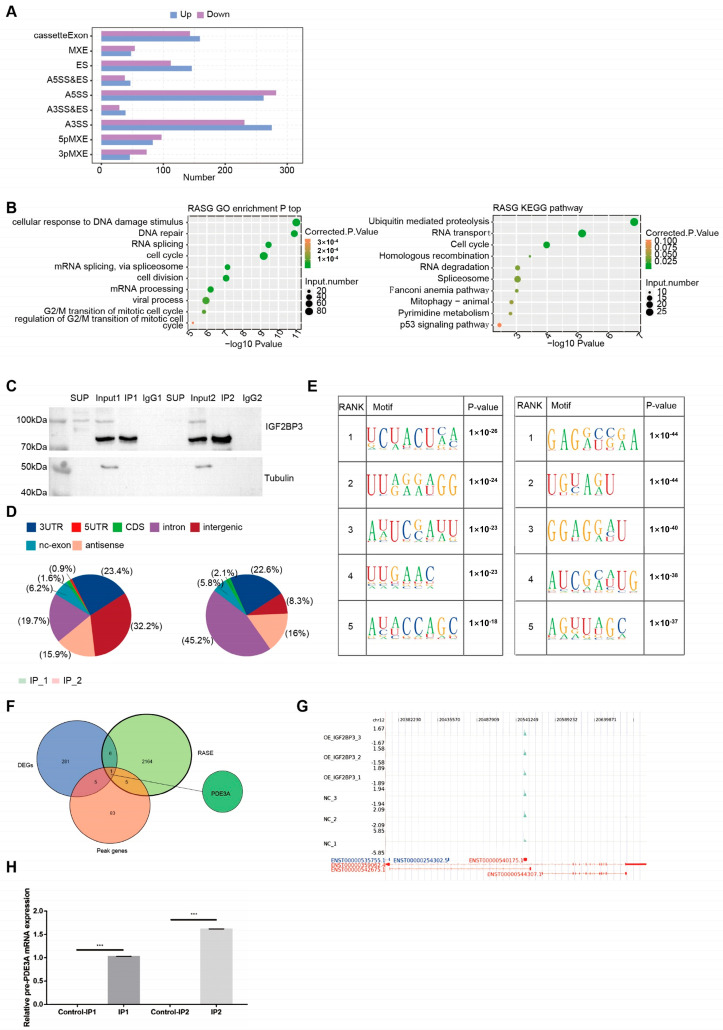
IGF2BP3 regulates gene alternative splicing in HTR-8/SVneo trophoblast cells: (**A**) Bar plot of the IGF2BP3 regulated alternative splicing event numbers by classifying them into nine types. (**B**) Bubble plot of the top 10 enriched GO terms for IGF2BP3 regulated alternative splicing genes. (**C**) Western blot analysis of IGF2BP3 immunoprecipitates. (**D**) Pie chart of the genomic distribution of IGF2BP3-bound peaks from the two biological replicates. (**E**) Motif enrichment analysis was conducted on these IGF2BP3-binding regions. (**F**) Venn diagram illustrating the overlap among DEGs, RASEs, and peak-associated genes. (**G**) Read distribution across the entire PDE3A locus. (**H**) RIP-qPCR verification of IGF2BP3 binding to the PDE3A gene. All data are expressed as mean ± SEM. *** *p* < 0.001.

To explore the involvement of IGF2BP3 in alternative splicing (AS) regulation, transcriptome sequencing was conducted to profile IGF2BP3-dependent AS events in HTR-8/SVneo trophoblast cells. A bar graph was generated to display the distribution of regulated RASE numbers, categorized into nine types, including cassette exon, MXE, ES, A5SS&ES, A5SS, A3SS&ES, A3SS, 5pMXE, and 3pMXE. In total, 2164 significantly altered AS events were identified (Figure 4A). Furthermore, a bubble plot illustrated the top ten enriched GO and KEGG pathways associated with IGF2BP3-regulated RASGs and their roles in RNA splicing and cell cycle processes (Figure 4B). Collectively, these findings demonstrate that IGF2BP3 plays a pivotal role in modulating AS in HTR-8/SVneo trophoblast cells.

Western blot analysis showed the IGF2BP3 immunoprecipitates (Figure 4C) and the pie chart showed the genomic distribution of IGF2BP3-bound peaks from the two biological replicates (Figure 4D) and the motif analysis results showed the enriched motifs from IGF2BP3-bound peaks from the two biological replicates (Figure 4E). To further investigate the relationship between IGF2BP3 binding and the regulation of AS, we performed overlap analysis of the DEGs from the overexpressed RNA-seq analysis, RASE and the peak genes from the RIP-seq analysis. The results showed that there was only one overlap gene, PDE3A (Figure 4F). As shown in Figure 4G, there are a large amount of IGF2BP3-bound peaks located in the exonic position of PDF3A. Moreover, the qPCR analysis data showed that the expression of pre-PDE3A is decreased significantly (Figure 4H), which indicates that in HTR-8/SVneo trophoblast cells AS of PDE3A is regulated by IGF2BP3 directly.

### 3.5. IGF2BP3 Targets and Stabilizes PDE3A3 mRNA

**Figure 5:**
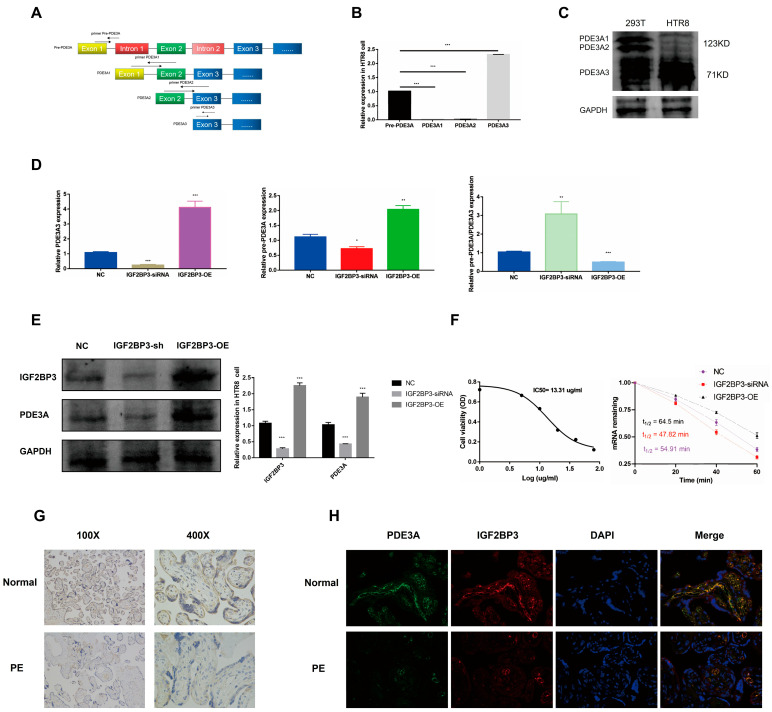
IGF2BP3 targets and stabilizes PDE3A3 mRNA: (**A**) Sketch map of the exons distribution of PDE3A isoforms. (**B**) RT-qPCR of the expression of PDE3A isoforms in HTR-8/SVneo trophoblast cells. (**C**) Western blotting of the expression of PDE3A isoforms in HTR-8/SVneo trophoblast cells and 293T cells. (**D**) RT-qPCR of PDE3A3 regulated by IGF2BP3. (**E**) Western blotting of the expression of PDE3A3 regulated by IGF2BP3. (**F**) Prolonged PDE3A mRNA half-life by overexpressing IGF2BP3 in HTR-8/SVneo trophoblast cells. (**G**) IHC staining of candidate gene PDE3A in PE placentas and normal control tissues. (**H**) IF staining of IGF2BP3 and PDE3A in PE placentas and normal control tissues. Scale bar, 100 µm. Data at the right are presented as mean ± SEM. * *p* < 0.05, ** *p* < 0.01, *** *p* < 0.001.

Figure 5A shows the three isoforms of PDE3A (PDE3A1, PDE3A2, and PDE3A3) depending on alternative transcriptions which have been identified in human myocardium. Literature has confirmed that the three isoforms control different physiologic responses through unique protein–protein interactions. qPCR analysis suggested that PDE3A3 is highly expressed specifically in HTR-8/SVneo trophoblast cells (Figure 5B). Western blotting assay also showed that PDE3A3 was verified as trophoblast-specific expressed protein because the expression of PDE3A3 was highly expressed in HTR-8/SVneo trophoblast cells but not 293T cells (Figure 5C). Since IGF2BP3, as an RNA binding protein, improved the stability of many target mRNAs, we then further aimed to elucidate the mechanisms underlying IGF2BP3 regulation of PED3A function. qPCR and Western blotting assay showed that the expression of PED3A was upregulated in IGF2BP3-overexpressed HTR-8/SVneo trophoblast cells (Figure 5D,E). Moreover, to examine the influence of IGF2BP3 overexpression on PDE3A3 mRNA stability, we used actinomycin D to stop mRNA transcription in IGF2BP3-overexpressed or IGF2BP3-knocked down HTR-8/SVneo trophoblast cells and measured the decay rates for PDE3A3 mRNA. The half-life of PDE3A3 mRNA decreased from 60.31 min to 47.82 min in the absence of IGF2BP3 (Figure 5F), supporting a role of IGF2BP3 in stabilizing PDE3A3 mRNA. Together, our findings supported that IGF2BP3 enhances the mRNA stability of PDE3A and PDE3A3 is a key target of IGF2BP3.

### 3.6. Knockdown of PDE3A Partially Damages the Promotion Effects of IGF2BP3 Overexpression

**Figure 6:**
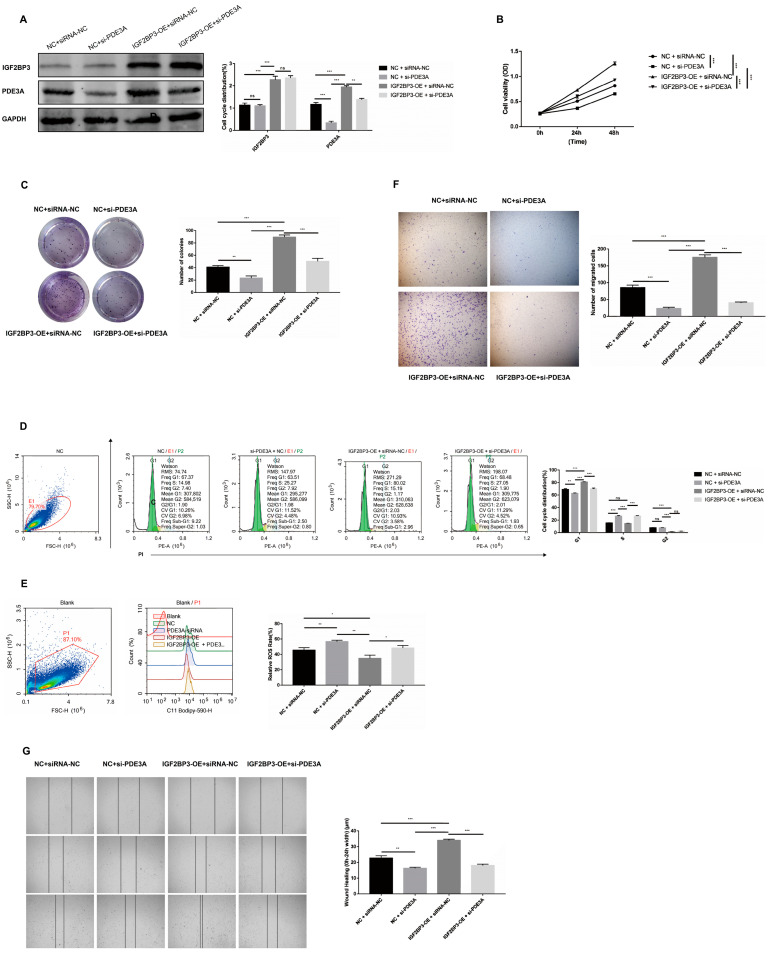
Knockdown of PDE3A partially damages the promotion effects of IGF2BP3 overexpression: (**A**) Western blotting of the expression of IGF2BP3 and PDE3A in HTR-8/SVneo trophoblast cells infected with silencing PDE3A, overexpressing IGF2BP3 plus silencing PDE3A, or negative control. (**B**) Cell proliferation was determined for HTR-8/SVneo trophoblast cells infected with silencing PDE3A, overexpressing IGF2BP3 plus silencing PDE3A, or negative control by CCK8 assay. (**C**) Colony formation assays were performed with HTR-8/SVneo trophoblast cells infected with silencing PDE3A, overexpressing IGF2BP3 plus silencing PDE3A, or negative control. (**D**) Cell cycle analysis was performed with HTR-8/SVneo trophoblast cells infected with silencing PDE3A, overexpressing IGF2BP3 plus silencing PDE3A, or negative control. (**E**) ROS activity was measured by C11-BODIPY 581/591 intensity in HTR-8/SVneo trophoblast cells infected with silencing PDE3A, overexpressing IGF2BP3 plus silencing PDE3A, or negative control. (**F**) Cell invasion was assessed using a Transwell assay in HTR-8/SVneo trophoblast cells following PDE3A knockdown, IGF2BP3 overexpression combined with PDE3A silencing, or negative control treatment. (**G**) Cell migration was evaluated by wound healing assay under the same conditions. All quantitative data are reported as mean ± SEM. * *p* < 0.05, ** *p* < 0.01, *** *p* < 0.001.

To investigate the functional link between IGF2BP3 and PDE3A in HTR-8/SVneo trophoblast cells, we first generated stable HTR-8/SVneo trophoblast cells with PDE3A knockdown and meanwhile overexpressed IGF2BP3 (Figure 6A). We found that knockdown of PDE3A led to the reduction in proliferating cells and numbers of survival colonies compared with IGF2BP3 overexpression in HTR-8/SVneo trophoblast cells (Figure 6B,C). Similar effects on cell cycle, ROS activity, and migration ability were also observed in HTR-8/SVneo trophoblast cells with PDE3A knockdown and IGF2BP3 overexpression (Figure 6D–G). These observations strongly suggest that IGF2BP3 regulates the AS of PDE3A, which mediates the function in HTR-8/SVneo trophoblast cells.

### 3.7. MiR-196a-5p -Mediated Post-Transcriptional Regulation of IGF2BP3

As an RNA-binding protein (RBP), IGF2BP3 plays a crucial role in regulating mRNA stability, localization, and translation. Additionally, IGF2BP3 itself is subject to post-transcriptional regulation by microRNAs. These microRNAs can bind to IGF2BP3 mRNA, leading to its degradation or translational repression, thereby modulating its expression and functional impact across various cellular contexts, including trophoblast development and pathological conditions such as preeclampsia.

To identify potential microRNA regulators of IGF2BP3, we utilized multiple target prediction databases, including miRDB, DIANA-microT, TargetScan, and StarBase. Furthermore, we analyzed differential gene expression in HTR8/SVneo cells through RNA sequencing (RNA-seq), identifying several candidate microRNAs, such as hsa-miR-204-5p, hsa-miR-211-5p, and hsa-miR-196a-5p. Based on both target gene predictions and RNA-seq results, we selected hsa-miR-196a-5p for further evaluation.

**Figure 7:**
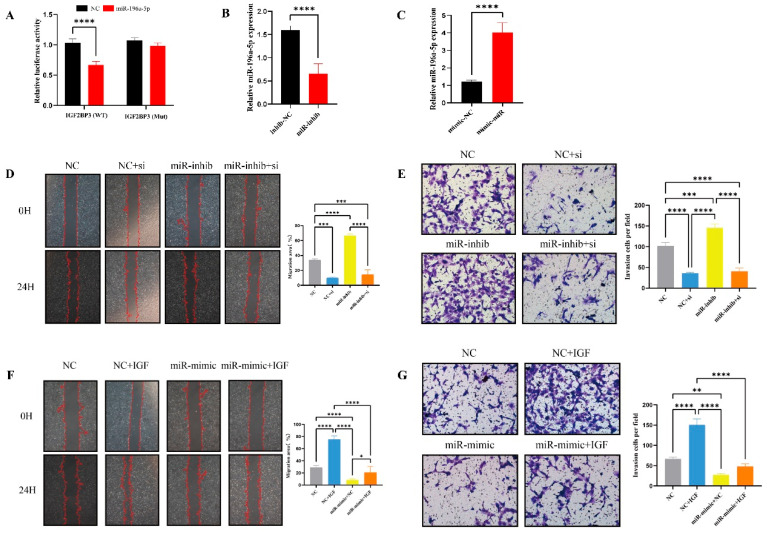
MiR-196a-5p suppresses HTR-8/SVneo cell invasion and migration: (**A**) Dual-luciferase reporter assay results demonstrating the specific targeting of miR-196a-5p to the wild-type (WT) IGF2BP3 3′ UTR, with significantly reduced activity compared to the mutant (Mut) and negative control (NC), indicating direct interaction. (**B**) Bar graph showing decreased miR-196a-5p expression following treatment with a specific inhibitor, confirming inhibitor efficacy. (**C**) Bar graph displaying increased miR-196a-5p expression upon introduction of miR-mimic compared to mimic-NC, validating effective overexpression. (**D**) Migration analysis via wound healing assay, depicting enhanced migration in cells treated with miR-196a-5p inhibitor (miR-inhib) and a reduction in migration upon IGF2BP3 knockdown (miR-inhib + si). Quantitative analysis confirms statistical significance. (**E**) Invasion assay results showing increased invasive capacity in miR-inhib-treated cells, which is notably decreased upon additional IGF2BP3 knockdown (miR-inhib + si). The changes are statistically significant. (**F**) Migration assay revealing decreased migration in cells overexpressing miR-196a-5p (miR-mimic), with partial restoration of migration following the addition of exogenous IGF2BP3 (miR-mimic + IGF), demonstrates the significance of the recovery. (**G**) Analysis of invasion capabilities post miR-196a-5p overexpression, showing reduced invasion in miR-mimic cells, with a partial recovery upon IGF2BP3 addition, still below the levels seen in NC + IGF group. * *p* < 0.05, ** *p* < 0.01, *** *p* < 0.001, **** *p* < 0.0001.

To verify the interaction between miR-196a-5p and IGF2BP3, a dual-luciferase reporter assay was performed in HEK-293T cells. Constructs containing either the wild-type (WT) or mutant (MUT) IGF2BP3 sequences were inserted into the pmirGLO luciferase vector. Experimental results revealed that the miR-196a-5p mimic significantly suppressed the luciferase activity of the WT-IGF2BP3 construct, while the mutant version showed no notable change (Figure 7A).

### 3.8. MiR-196a-5p Suppresses HTR-8/SVneo Cell Invasion and Migration

To investigate the potential roles of miR-196a-5p in trophoblast invasion and migration, we treated HTR-8/SVneo cells with either the hsa-miR-196a-5p inhibitor (hsa-miR-196a-5p-inhib) or its corresponding negative control (hsa-miR-196a-5p-inhiNC), and qRT-PCR analysis confirmed a significant reduction in miR-196a-5p levels. Functional assays demonstrated that inhibiting miR-196a-5p led to a marked increase in cell invasion and migration compared to the control group (Figure 7B–E).

To further explore its functional significance, we assessed the impact of miR-196a-5p overexpression. We overexpressed miR-196a-5p in HTR-8/SVneo cells using an miR-196a-5p mimic (hsa-miR-196a-5p-mimic) and compared the results with its corresponding negative control (hsa-miR-196a-5p-mimicNC), and qRT-PCR analysis verified a significant upregulation of miR-196a-5p expression. Invasion and migration assays showed that overexpression of miR-196a-5p significantly reduced the invasive and migratory abilities of HTR-8/SVneo cells compared to the control group, further supporting its inhibitory role in trophoblast cell motility (Figure 7C,F,G).

### 3.9. miR-196a-5p Negatively Regulates IGF2BP3 and PDE3A Expression

**Figure 8:**
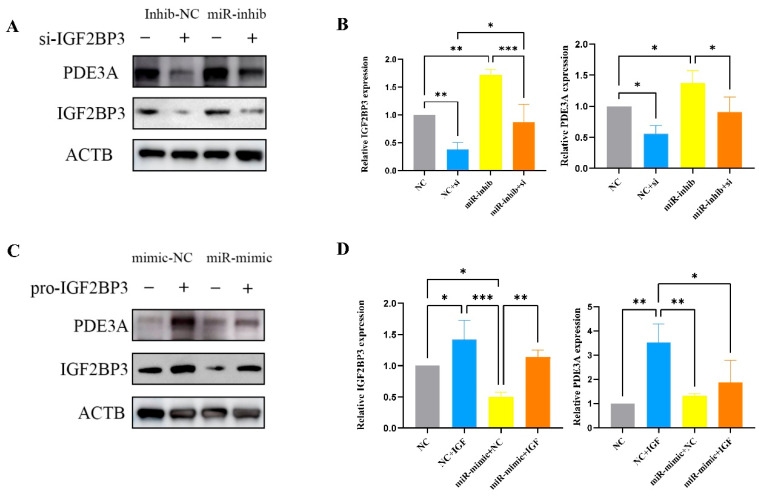
(**A**,**B**) Show the increase in IGF2BP3 and PDE3A protein and mRNA levels following miR-196a-5p inhibition, and their subsequent decrease upon siRNA-mediated IGF2BP3 knockdown. These changes are confirmed by Western blot and qPCR, with significant expression alterations noted. (**C**,**D**) Depict the reduction in IGF2BP3 and PDE3A protein and mRNA levels after miR-196a-5p overexpression, which are partially restored with the addition of exogenous IGF2BP3. Both Western blot and qPCR analyses illustrate these dynamics, highlighting the regulatory role of miR-196a-5p and the compensatory effect of added IGF2BP3 (* *p* < 0.05, ** *p* < 0.01, *** *p* < 0.001).

To investigate whether IGF2BP3 is directly regulated by miR-196a-5p, HTR-8/SVneo cells were treated with an miR-196a-5p inhibitor, and subsequent changes in IGF2BP3 and PDE3A expression levels were assessed. qRT-PCR analysis revealed a significant upregulation of IGF2BP3 and PDE3A following miR-196a-5p inhibition (Figure 8B). In contrast, IGF2BP3 siRNA transfection effectively reduced the expression of both IGF2BP3 and PDE3A (Figure 8A,B). Functional analyses demonstrated that IGF2BP3 knockdown inhibited HTR-8/SVneo cell invasion and migration (Figure 7D,E). Notably, IGF2BP3 siRNA also attenuated the pro-invasive and pro-migratory effects induced by miR-196a-5p inhibition, indicating that IGF2BP3 is a critical mediator of miR-196a-5p function (Figure 7D,E). Parallel experiments confirmed corresponding changes in IGF2BP3 and PDE3A protein levels (Figure 8A).

To further elucidate the regulatory interplay between miR-196a-5p and IGF2BP3 in trophoblast invasion and migration, an miR-196a-5p mimic was introduced into HTR-8/SVneo cells. Transfection with the miR-196a-5p mimic significantly increased miR-196a-5p expression, as confirmed by qRT-PCR (Figure 7C). To determine whether IGF2BP3 modulates trophoblast cell behavior by acting as a competing endogenous RNA (ceRNA) for miR-196a-5p, a series of rescue experiments were performed. Co-transfection of IGF2BP3 with miR-196a-5p mimics restored IGF2BP3 and PDE3A expression, which had been suppressed by miR-196a-5p overexpression (Figure 8D). Moreover, IGF2BP3 overexpression not only promoted HTR-8/SVneo cell invasion and migration (Figure 7F,G) but also reversed the inhibitory effects of miR-196a-5p mimics on these cellular processes. Collectively, these findings highlight miR-196a-5p as a negative regulator of trophoblast invasion and migration, exerting its effects through direct targeting of IGF2BP3 and PDE3A.

## 4. Discussion

Accumulating evidence suggests that dysregulation of mRNA metabolism and RNA editing plays a pivotal role in the pathogenesis of preeclampsia (PE) [14]. In this study, RNA sequencing (RNA-seq) data were analyzed to investigate alternative splicing (AS) events in PE patient samples. Our findings identified IGF2BP3, a key RNA-binding protein (RBP), as a regulator of AS processes associated with PE. In vitro assays demonstrated that IGF2BP3 expression was significantly downregulated in PE placental tissues. Functional studies further revealed that IGF2BP3 overexpression promoted the proliferation and migration of HTR-8/SVneo trophoblast cells. Moreover, using iRIP-seq and Actinomycin D assays, we confirmed that IGF2BP3 directly binds to PDE3A mRNA and enhances its stability, thereby regulating alternative splicing in trophoblast cells.

Previous studies have reported that IGF2BP3 knockdown suppresses trophoblast invasion and migration via the PI3K/AKT signaling pathway [13]. Consistent with these findings, we observed decreased IGF2BP3 expression in PE tissues compared with normal placental tissues. Overexpression of IGF2BP3 not only enhanced proliferation, colony formation, invasion, and migration of HTR-8/SVneo trophoblast cells but also reduced reactive oxygen species (ROS) activation, further highlighting its potential as a therapeutic target for PE.

AS is a crucial post-transcriptional mechanism that generates multiple mRNA isoforms from a single gene, significantly contributing to transcriptome and proteome diversity [13]. Extensive research has established that AS plays a critical role in tumorigenesis by modulating mRNA metabolism [21]. However, its role in PE remains largely unexplored. One notable example is sFLT1, a soluble VEGF receptor variant, which is upregulated in PE due to an AS event and serves as a hallmark of the disease [22]. In this study, we identified IGF2BP3 as a key regulator of AS events during PE progression and validated its functional RNA targets in vitro. IGF2BP3 has been reported to interact with m6A-modified RNAs, influencing their stability and alternative splicing [23]. Previous studies have demonstrated that IGF2BP3 expression is reduced in PE placental tissues and that its knockdown inhibits trophoblast migration and invasion [13]. Additionally, IGF2BP3 has been implicated in lung tumorigenesis through AS regulation of PKM [12]. Moreover, IGF2BP3 interacts with nuclear RNA 7SK, modulating its stability and leading to aberrant splicing events, ultimately causing developmental defects in zebrafish embryos [24]. Despite these insights, the specific RNA targets of IGF2BP3 in trophoblast cells remain largely unexplored.

To systematically identify IGF2BP3-associated mRNAs, we performed iRIP-seq in HTR-8/SVneo trophoblast cells. Our analysis revealed that IGF2BP3 overexpression significantly altered gene expression and AS profiles. Notably, PDE3A mRNA was identified as a direct IGF2BP3 target. RNA stability assays demonstrated that IGF2BP3 overexpression prolonged the half-life of PDE3A mRNA, leading to its upregulation. Further in vitro experiments confirmed that IGF2BP3 binding enhances PDE3A mRNA stability, thereby promoting the proliferation and migration of trophoblast cells.

PDE3A encodes a member of the cGMP-inhibited cyclic nucleotide phosphodiesterase (cGI-PDE) family, which plays a critical role in various cellular processes by regulating cyclic nucleotide signaling [25]. Originally cloned from human myocardial tissues, PDE3A is also expressed in vascular smooth muscle cells, platelets, oocytes, kidneys, and the cervix [26]. Three PDE3A isoforms—PDE3A1, PDE3A2, and PDE3A3—have been identified, sharing identical sequences except for their terminal regions. Recent studies have revealed significant differences in protein interactions among these isoforms [27]. Notably, PDE3A3 is exclusively expressed in placental tissues, whereas PDE3A1 is predominantly found in myocardial cells, a finding that aligns with our results [28]. Our data further confirmed that PDE3A3 is highly expressed in HTR-8/SVneo trophoblast cells, whereas PDE3A1/2 is primarily expressed in HEK293T cells. Previous research has demonstrated that PDE3A inhibition modulates cell cycle progression via the cAMP/PKA/MAPK signaling pathway in vascular smooth muscle cells and oocytes [29]. In this study, we found that PDE3A knockdown suppressed trophoblast proliferation and migration and induced G1-phase cell cycle arrest, suggesting that PDE3A plays a crucial role in cell cycle regulation.

Bioinformatics analysis predicted that IGF2BP3 is directly inhibited by miR-196a-5p, a microRNA transcribed from the HOXC gene cluster, known to play critical roles in cell proliferation, differentiation, apoptosis, and migration across various tissues [28]. Dysregulation of miR-196a-5p has been implicated in the progression of multiple cancers and pregnancy-related disorders. This prediction was experimentally validated through luciferase reporter assays, qRT-PCR, and Western blotting. Furthermore, IGF2BP3 knockdown via siRNA mimicked the effects of miR-196a-5p overexpression, significantly reducing trophoblast invasion and migration. Conversely, reintroducing IGF2BP3 expression partially rescued the inhibitory effects of miR-196a-5p on trophoblast motility. Notably, miR-196a-5p was highly expressed in PE placental tissues, further supporting its role in PE pathogenesis. These findings collectively indicate that miR-196a-5p suppresses cytotrophoblast invasion and migration by directly targeting IGF2BP3.

MiRNAs play critical roles in cell proliferation, differentiation, and apoptosis, acting as either oncogenic or tumor-suppressive regulators. Extensive research has demonstrated that miRNAs regulate trophoblast proliferation, migration, and apoptosis during pregnancy [30]. Several studies have identified miR-196a-5p as an oncogenic factor in various cancers, promoting disease progression [31]. Interestingly, miR-196a-5p expression was significantly downregulated in placenta accreta spectrum (PAS) but upregulated in PE placenta [32]. Consistent with these findings, our study revealed that miR-196a-5p is upregulated in PE and suppresses trophoblast proliferation and migration by downregulating IGF2BP3, thereby contributing to PE pathogenesis, along with the elucidation of the miR-196a-5p/IGF2BP3/PDE3A regulatory axis, providing novel mechanistic insights into the pathogenesis of preeclampsia (PE).

However, certain limitations should be acknowledged. Firstly, the current findings are primarily based on in vitro cellular models, lacking validation in in vivo animal studies and clinical patient cohorts, which limits the translational applicability of the results. Secondly, while focused on PDE3A as a key downstream effector of IGF2BP3, we acknowledge the emerging significance of apoptotic and autophagic regulators such as BCL-2 and Beclin-1 in preeclampsia and HELLP syndrome [33]. Given the emerging evidence linking IGF2BP3 to cell survival and stress response pathways, future studies might explore its role in modulating apoptosis and autophagy in the context of PE. Additionally, the heterogeneity of PE subtypes and the complexity of tropho-blast-immune-endothelial interactions warrant further investigation to delineate the precise contribution of IGF2BP3 in PE pathogenesis.

In conclusion, our study identifies IGF2BP3 as a novel regulator of alternative splicing and mRNA stabilization in trophoblast cells, contributing to PE progression through its downstream target PDE3A. These findings provide a foundation for future research into the post-transcriptional regulatory networks in PE and highlight IGF2BP3 as a potential therapeutic target for the treatment of hypertensive pregnancy disorders.

## Data Availability

The original contributions presented in this study are included in the article, and further inquiries can be directed to the corresponding authors.

## Abbreviations

The following abbreviations are used in this manuscript:

PE	Preeclampsia
AS	Alternative splicing
IGF2BP3	Insulin-like growth factor 2 mRNA-binding protein 3
RBPs	RNA-binding proteins
RIP-seq	RNA immunoprecipitation sequencing
PDE3A	Phosphodiesterase 3A
RNA-seq	RNA sequencing
DEGs	Differentially Expressed Genes
ES	exon skipping
A5SS	alternative 5′ splice site
A3SS	alternative 3′s splice site
MXE	mutually exclusive exons
5pMXE	mutually exclusive 5′UTRs
3pMXE	mutually exclusive 3′UTRs
sFLT1	Soluble fms-like tyrosine kinase-1
VEGF	Vascular Endothelial Growth Factor
PKM	Pyruvate Kinase M
GO	Gene Ontology
KEGG	Kyoto Encyclopedia of Genes and Genomes
ASEs	alternative splicing events
ROS	Reactive Oxygen Species
RASEs	regulated alternative splicing events

## Appendix A

**Table A1:** qPCR primers sequence.

IGF2BP3-F	5′-ACGAAATATCCCGCCTCATTTAC-3′
IGF2BP3-R	5′-GCAGTTTCCGAGTCAGTGTTCA-3′
PDE3A-F	5′-CCACGGCCTCATTACCGAC-3′
PDE3A-R	5′-TTGCTCACGGCTCTCAAGG-3′
PDE3A1-F	5′-TGGAGACCTTACCTGGCGTA-3′
PDE3A1-R	5′-TGAATGCCCCATGAGCTGTT-3′
PDE3A2-F	5′-AAAGCAAAGTCGACCAGATGA-3′
PDE3A2-R	5′-GGTCTCAGGAGCATAGGTGC-3′
PDE3A3-F	5′-AAATCGTGGAGCTTCTACTGGG-3′
PDE3A3-R	5′-GTGACGGGATTCACTCTGGG-3′
GAPDH-F	5′-AGAAGGCTGGGGCTCTCATTG-3′
GAPDH-R	5′-AGGGGCCATCCACAGTCTTTC-3′
Beta-actin-F	5′-AGCGAGCATCCCCCAAAGTT-3′
Beta-actin-R	5′-GGGCACGAAGGCTCATCATT-3′
